# Chemical structure and gelation characteristics of a purified gel derived from sword beans (*Canavaliagladiata*)

**DOI:** 10.1016/j.heliyon.2024.e24900

**Published:** 2024-01-17

**Authors:** Yasuhiro Arii, Kaho Nishizawa

**Affiliations:** a Department of Innovative Food Sciences, School of Food Sciences and Nutrition, Mukogawa Women's University, Nishinomiya, Hyogo, 663-8558, Japan; b Research Institute for Nutrition Sciences, Mukogawa Women's University, Nishinomiya, Hyogo, 663-8558, Japan; c Department of Food Sciences and Human Nutrition, Faculty of Agriculture, Ryukoku University, Otsu, Shiga, 520-2194, Japan

**Keywords:** *Canavalia gladiata*, Gel, Starch

## Abstract

Herein, a new method was developed to obtain a crude extract from sword beans at a higher extraction efficiency. The crude extract formed a gel at 8 °C, which melted at 70 °C, and lyophilization of the purified gel produced a powder that could be dissolved in distilled water at a concentration of 7 % (w/w) or less. A 3 % powder solution gelled at 12 °C and melted at 60 °C. The infrared spectrum of the gel powder was consistent with that of starch. Furthermore, a 4-aminobenzoic acid ethyl ester-labeling analysis revealed that glucose was the constituent sugar in the powder, and the powder solution reacted strongly in a starch–iodine test. These observations confirmed that the gelling substance was starch. However, the melting and gelling temperatures were dissimilar to those of other starches frequently used in the food industry. Thus, our results provide valuable information for using sword bean starch as a novel food material.

## 1 Introduction

Sword beans (*Canavalia gladiata*) are edible leguminous plants originating from Asia and Africa [[1], [2], [3]]. The average yield of sword beans per unit area is comparable to that of soybeans [2], and the sword bean plant is relatively resistant to pests and diseases [4]. Dried sword beans contain 26 % protein, 3 % fat, and 62 % carbohydrates [5]. Thus, sword beans demonstrate great potential as food materials from both agricultural and nutritional standpoints. However, to date, sword beans remain underutilized.

The present authors previously developed a method to prepare three crude sword bean extracts [6]. Of these, only one extract, which was prepared by boiling a suspension of soaked ground beans in distilled water (DW), gelled upon incubation at below 10 °C, and the resulting gel melted at above 65 °C [7]. Notably, this extract remained a liquid at room temperature, unlike other commercially available gelling food materials. Sodium dodecyl sulfate-polyacrylamide gel electrophoresis analysis suggested that the gelling substance in the extract was very unlikely to be a protein [6]; thus, we presumed it to be a carbohydrate [7]. As dry sword beans contain approximately 40 % starch [8], the carbohydrate is likely to be a starch. However, the crude extract only reacted slightly in a starch–iodine test [7]. Interestingly, the ability of the extract to gel at below room temperature (25 °C) and melt at high temperatures is highly useful in food processing. Nevertheless, the extract requires further purification, and its chemical structure and physicochemical properties should be clarified.

This study aimed to determine the chemical structure and physicochemical properties of the gelling substance. We also attempted to improve the efficiency and stability of the extraction method. We further purified a gelling substance from the extract and dried it to a powder. Following this, the solubility, chemical structure, and melting and gelling temperatures were analyzed. In addition, the purified gel powder solution was subjected to a starch–iodine reaction. Although more detailed studies are required before the extract can be used as a new food material, our results will facilitate quantitative studies of the sword bean gel as a new food material.

## 2 Experimental

### 2.1 Materials

Dried white sword beans were purchased from Morika Kometen (Nara, Japan). General chemical reagents, soluble starch (amylodextrin produced from hydrolysis of potato starch, product code 191–03985), corn starch (product code 193–09925), and wheat starch (product code 193–13215) were purchased from FUJIFILM Wako Pure Chemical Corporation (Osaka, Japan). Potato starch was purchased from Hokuren (Sapporo, Japan). A 4-aminobenzoic acid ethyl ester (ABEE)-labeling kit and a standard mixture (monosaccharide mixture-11 containing d-galactose, d-mannose, d-glucose, l-arabinose, d-ribose, d-xylose, N-acetyl-d-mannosamine, N-acetyl-d-glucose, N-acetyl-d-galactose, l-fucose, and rhamnose) were purchased from MGC Woodchem Corporation (Tokyo, Japan).

### 2.2 Preparation of crude extracts using different methods

Two methods were used to prepare the crude extracts, with major differences in the processing of dry beans and the preparation of the heated suspension. In the aforementioned previous method [7], dry beans were soaked in 10 vol (v/w) of DW at 20 °C for 18 h. The soaked beans were drained and ground on ice for 5 min in eight volumes (v/w) of DW using a hand blender (SB-77JBSTRW, Cuisinart, Stamford, CT, USA). The crude suspension was boiled with gentle stirring on a heated stir plate (RET control-visc, IKA, Staufen, Germany) for 3 min. Meanwhile, in the proposed extraction method, dry beans were milled using a grinder (Force Mill, Y–308B, Osaka Chemical Co., Ltd., Osaka, Japan) for 2 min. The bean flour was suspended in eight volumes (v/w) of DW and stirred at 500 rpm and room temperature (25 °C) for 1 h. Finally, the suspension was autoclaved at 121 °C for 15 min.

After dropping to 60 °C, each hot suspension was squeezed through a cotton cloth to separate the filtrate from the solid residue. The filtrate was further centrifuged at 9100×*g* at 20 °C for 10 min, and the supernatant was used as the crude extract. The effect of different heating methods on extraction was compared by evaluating the gelatinization of both crude extracts.

### 2.3 Evaluation of gelation

Gelation was evaluated as previously described [7]. Samples were incubated at 20 °C or 4 °C for 2 days and separated into the supernatant and precipitate (gel) by centrifugation at 9100×*g* at 20 °C for 10 min. The initial sample and obtained wet gel were weighed using an electronic balance (HR-120, A&D Company, Tokyo, Japan). The gelation efficiency was calculated as the weight percentage of the wet gel relative to the initial sample, and the data are presented as the mean ± standard deviation calculated over three independent replicates.

### 2.4 Preparation of purified gel powder

The crude extract obtained through the new extraction method (grinding–soaking–autoclaving) was gelled at 4 °C, following which the gel and residual liquid were separated from each other as described above. The gel was washed in five volumes (v/w) of DW for more than three times. After each washing, the gel was weighed again, and the degree of purification was evaluated using the weight change of the wet gel and the reducing sugar content in the supernatant (see Section 2.6 below). Finally, the triply purified samples were lyophilized and stored at −25 °C.

### 2.5 Dissolution of gel powder and starches

The obtained gel powder and three reference starches (soluble starch, corn starch, and wheat starch) were dispersed in DW at various concentrations, incubated at 100 °C for 1 h, and autoclaved at 121 °C for 15 min.

### 2.6 Measurement of the reducing sugar content

The reducing sugar content was measured using the Somogyi–Nelson method [9], with some minor modifications. The sample was mixed with an equal volume of the Somogyi solution (Wako Pure Chemical Industries), heated in a boiling bath for 10 min, and cooled on ice for 5 min. The cooled sample was added to an equal volume of the Nelson solution (Wako Pure Chemical Industries) and mixed together. The mixture was diluted with 20 vol of DW. After incubation at room temperature (25 °C) for 15 min, the absorbance was measured at 500 nm. The reducing sugar content was determined from a standard curve established using glucose solutions at 0–150 μg/mL.

### 2.7 Analyses of the gelation and gel melting temperatures

The gelation and gel melting temperatures were measured according to our previous method [7]. To determine the gelation temperature, the crude extract and purified gel powder solution (3 %) were incubated at various temperatures for 2 days, and their gelation efficiencies were evaluated. To determine the gel melting temperature, gels produced by incubation at 4 °C for 2 days were heated to various temperatures and kept for 5 min. The data are presented as the average of three independent experiments.

### 2.8 Analysis of sugar composition

The sugar composition was determined according to a previous method [10], with some modifications. The gel powder and starch were hydrolyzed with trifluoroacetic acid (TFA) and fluorescence labeled using an ABEE-labeling kit as described below. The gel powder, starch, and glucose were dissolved separately in DW at 40 μg/mL. The solution was mixed with an equal volume of 8 M TFA, followed by heating at 100 °C for 3 h, and the mixture was dried under reduced pressure (60 kPa) at room temperature and 3250 rpm for 20 min in a centrifuge concentrator (CVE-1100, EYELA, Tokyo, Japan). The dried sample was dissolved in two volumes of isopropanol and dried again as described above. Next, the dried sample was dissolved in an equal volume of DW and then mixed with two volumes of the ABEE-labeling solution. After heating at 80 °C for 1 h, the mixture was combined with four volumes of DW and four volumes of chloroform and separated into a water phase and an organic phase by centrifugation at 5200×*g* for 10 s. The aqueous phase was injected into a reversed-phase C18 column (TSK gel, ODS-120H, 4.6 mm i.d. × 75 mm length, Tosoh, Tokyo, Japan) at a flow rate of 1.0 mL/min and 30 °C for 50 min using high-performance liquid chromatography (HPLC, LC-20A, Shimadzu, Kyoto, Japan). Fluorescence monitoring was conducted at an excitation wavelength of 305 nm and emission wavelength of 360 nm (RF-20A, Shimadzu, Kyoto, Japan). The mobile phase was a potassium borate buffer (0.2 M, pH 8.9) containing 7 % acetonitrile. A TFA solution of 0.02 % in 50 % acetonitrile was used for washing.

### 2.9 Attenuated total reflection Fourier transform infrared spectroscopy (ATR FT-IR)

The IR spectra were recorded using an ATR FT-IR spectrometer (IR Tracer-100, Shimadzu, Kyoto, Japan) at room temperature (25 °C). Each spectrum was based on 16 scans in the region between 4000 and 500 cm^−1^ at a resolution of 2 cm^−1^.

### 2.10 Starch–iodine test

The starch–iodine test was conducted as previously described [11], with some modifications. A reagent comprising 1 % potassium iodide and 0.25 % iodide was added to 40 vol of the test sample.

### 2.11 Statistical analysis

To compare the efficiency of the two extraction methods, significant differences were determined to examine the effects of heating methods using one-way analysis of variance and Tukey's honestly significant difference test using the KaleidaGraph software (ver. 5.0, Synergy, PA, USA). To clarify that the gelling temperature of the sword bean gel indeed differs from those of potato, wheat, and corn starches, significant differences were determined to examine the effects of heating methods using one-way analysis of variance and Tukey's honestly significant difference test using the same software. The student's *t*-test was used to compare the peak areas to confirm that the sword bean gel is a starch using the same software. The differences were considered statistically significant at *p* < 0.05.

## 3 Results and discussion

### 3.1 Improved method for preparing the crude extract

**Fig. 1:**
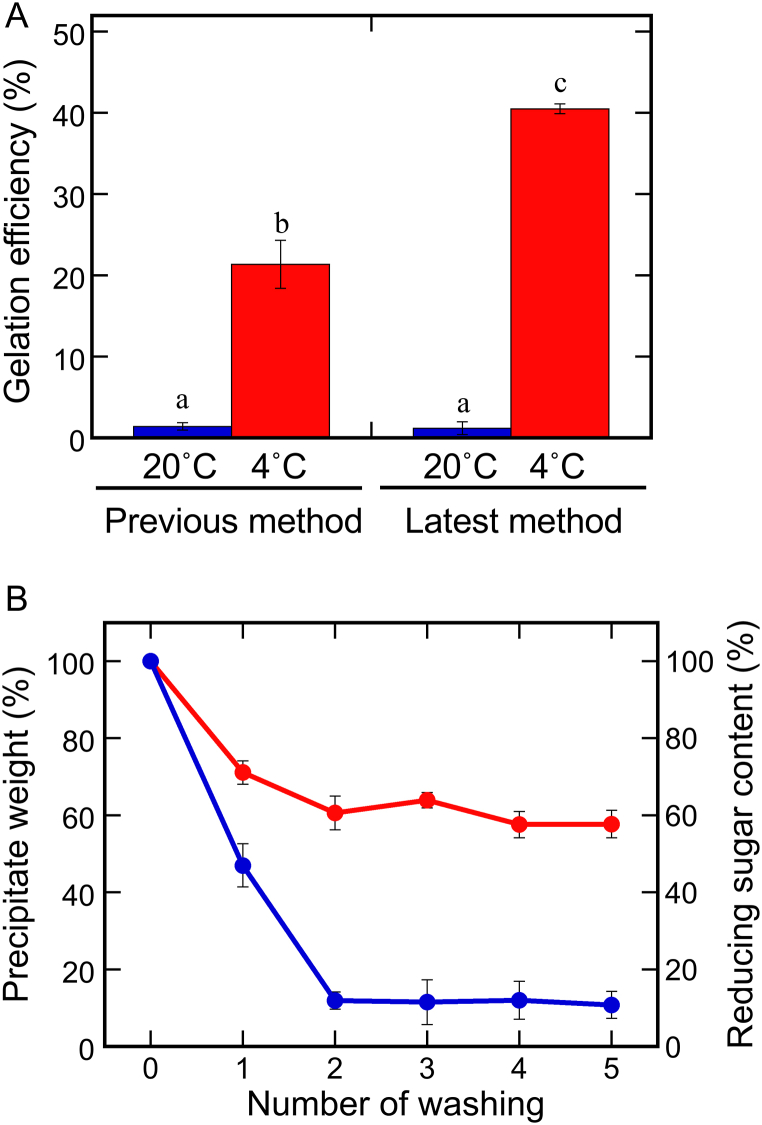
Extraction and purification of the gelling substance. (A) Comparison of the gelation efficiencies of the crude extracts prepared using two different methods (“Previous” and “Latest”). The extracts were incubated at 20 °C (blue bars) or 4 °C (red bars) for 2 days to determine the gelation efficiencies. Data are presented as the mean ± standard deviation of three independent replicates. The statistical significance of differences was determined by one-way analysis of variance and Tukey's honestly significant difference test. Different letters indicate statistically significant differences (*p* < 0.05). (B) Purification of the gel derived from sword bean extract. The prepared crude gel was washed in DW for five times. Red circles (left *y*-axis) indicate the changes in the wet precipitate weight, and blue circles (right *y*-axis) indicate the reducing sugar content of the supernatant. Data are presented as the mean ± standard deviation of three independent replicates. (For interpretation of the references to color in this figure legend, the reader is referred to the Web version of this article.)

The gelation efficiency and stability were compared between the previous and latest methods (Fig. 1A). The gelation efficiencies of the resultant crude extract were 1.2 ± 0.8 % at 20 °C and 40.5 ± 0.6 % at 4 °C. The previous extraction method (boiling a suspension prepared by grinding soaked beans) presented gelation efficiencies of 1.3 ± 0.5 % at 20 °C and 21.4 ± 3.0 % at 4 °C. The gelation efficiency of each extract was lower at 20 °C than that at 4 °C, and no difference was observed between the two extracts (*p* = 0.998). However, the proposed method demonstrated an almost doubled gelation efficiency at 4 °C, compared to the previous method (*p* < 0.001); hence it is a superior method for extracting gelling substances from sword beans.

The water-binding capacity of sword bean starch has been reported to increase with an increase in the heating temperature [8]. Therefore, we attribute the higher efficiency of the proposed extraction method to the higher water-binding capacity under the more severe heating conditions. Adebowale et al. (2006) proposed an extraction method for sword bean starch using highly alkaline sodium hydroxide [8]. By contrast, our extraction method involved much milder conditions (high temperature and pressure in DW and no added chemicals), making the process greener and the extracted starch safer for use in industrial applications. Based on these results, the gel prepared using the proposed method was used in the subsequent experiments.

### 3.2 Purification of the gel

To prepare the purified gel, the crude extract was incubated at 4 °C for 2 days, and the formed gel was repeatedly washed with DW (Fig. 1B). After washing twice, the reducing sugar content (blue circles) and gel weight (red circles) were approximately 12 % and 60 % of their initial values, respectively. A third washing attempt did not change either value significantly. Thus, the gels in the following experiments were purified by washing three times with DW. Finally, the thrice-washed gels were lyophilized and ground into a powder (gel powder). The weight of the wet purified gel was approximately 65 % that of the crude gel (Table 1). Overall, approximately 0.14 g of gel powder was obtained from 1.00 g of dried sword bean flour (Table 1). The preparation of such a purified gel allowed a quantitative analysis and the identification of the chemical structures therein. This yield was lower than that reported by Adebowale et al. (31 %) [8]. However, Adebowale et al. used room-temperature air drying, whereas we used freeze drying; thus, the two studies are not exactly comparable [8]. Notably, the starch yield may be improved by increasing the amount of DW added during extraction.

**Table 1:** Weight changes in each step of sword bean extraction and purification.

Purification stage	Weight (g)	Weight ratio
Sword bean flour	4.86 ± 0.04	
Crude extract	22.41 ± 1.09	4.61^a^
Crude gel	10.73 ± 0.59	2.21^a^
Washed gel	6.98 ± 0.68	1.44^a^ (0.65^b^)
Gel powder	0.67 ± 0.06	0.14^a^

a The ratio was calculated by dividing the sample weight at a given stage by the initial weight of sword bean flour.

b The ratio was calculated by dividing the sample weight after the third washing by the initial weight of crude gel. It indicates the proportion of residual after the washing steps.

### 3.3 Preparation and gelation of the purified gel powder solution

**Fig. 2:**
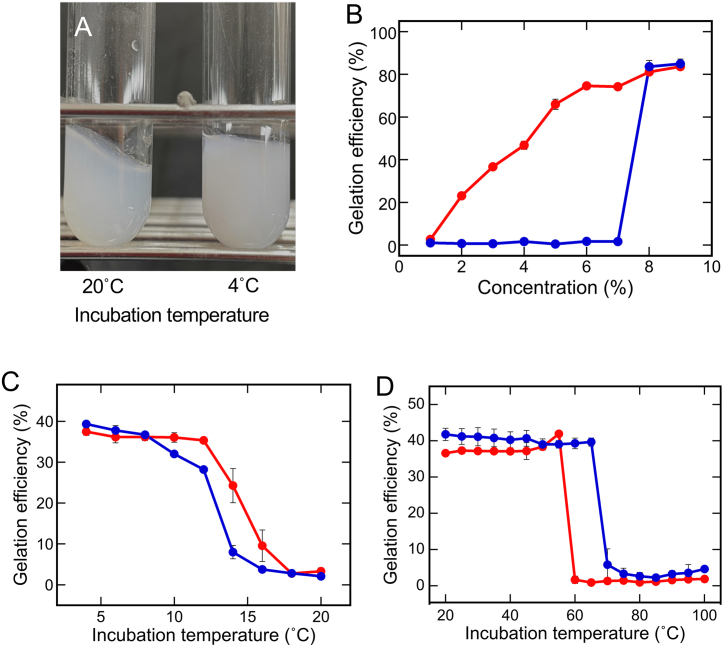
Physicochemical properties of the purified gel powder. The gel powder solution was incubated at 20 °C or 4 °C for 2 days at various concentrations. (A) Photographs of the gel sample obtained after incubating the 3 % gel powder solution, with the test tube inclined at 45°. (B) Gelation efficiency at 20 °C (blue circles) and 4 °C (red circles). Data are presented as the mean ± standard deviation of three independent replicates. (C) Gelation temperatures and (D) gel melting temperatures of the crude extract (blue circles) and purified gel powder solution (red circles). Data are presented as the mean ± standard deviation of three independent replicates. (For interpretation of the references to color in this figure legend, the reader is referred to the Web version of this article.)

The gel powder was insoluble in DW at room temperature. Thus, it was suspended in DW at a concentration of 3 % w/w and incubated at 100 °C for 1 h. The suspension was further autoclaved at 121 °C for 15 min to obtain a purified gel powder solution. The solution gelled at 4 °C but not at 20 °C (Fig. 2A). This gel powder solution had similar melting and gelling temperatures to those of the crude extract in our previous study [7].

For gel powder solutions at various concentrations (1–9%), their gelation efficiencies at 4 °C increased linearly from 1 % to 6 %, reaching 74.6 % (Fig. 2B), but not at 7 % or higher concentrations (red circles). When incubated at 20 °C, solutions at concentrations of 1–7% remained in the liquid form, while those above 8 % gelled with an efficiency similar to the case at 4 °C (blue circles). These results indicate that the gel powder could be dissolved in DW up to a concentration of 7 %, and the gelation efficiency is linear to the concentration up to 6 %. The ability to determine the solubility limits of the gel powder and concentration range to maintain linearity is important for basic and applied research in the future.

### 3.4 Comparison of the melting and gelling temperatures of the crude extract and gel powder solution

The gelation (Fig. 2C) and melting temperatures (Fig. 2D) were compared between the crude extract (blue circles) and gel powder solution (red circles), both prepared using the latest method. According to Fig. 1A, the gelation efficiency of the crude extract was approximately 40 %. To make the comparison as quantitative as possible, we chose the 3 % gel powder solution because it exhibits a gelation efficiency similar to the crude extract based on the results in Fig. 2B. In terms of the gelation temperature (Fig. 2C), the gel powder solution began to gel below 16 °C and almost completely gelled below 12 °C (red circles). By contrast, the crude extract began to gel below 14 °C, almost completely gelled below 8 °C (blue circles), and its gelation efficiency increased slightly when the temperature dropped from 12 °C to 8 °C. To determine the melting temperature, gels were prepared from the crude extract and 3 % gel powder solution by incubating at 4 °C for 2 days. Then, the gels were incubated at various temperatures from 20 to 100 °C to compare their melting temperatures (Fig. 2D). Gels prepared from the crude extract and gel powder solution almost completely melted at 70 °C (blue circles) and 60 °C (red circles), respectively. These differences in the gelation and melting temperatures could be attributed to the removal of impurities. In particular, impurities in the crude extract would hinder the interaction between starch molecules to lower the gelling temperature, whereas interactions between the starch and trapped impurities would tend to raise the melting temperature of the gel. However, the molecular mechanism underlying these unique gelling and melting temperatures remains unclear, and thus, we do not know the real reason for the temperature changes caused by purification. We plan to further analyze the gel structure at the molecular level, as the present study enabled a quantitative treatment of the purified gel, as shown in Fig. 2B.

### 3.5 Chemical structure of the gel powder

**Fig. 3:**
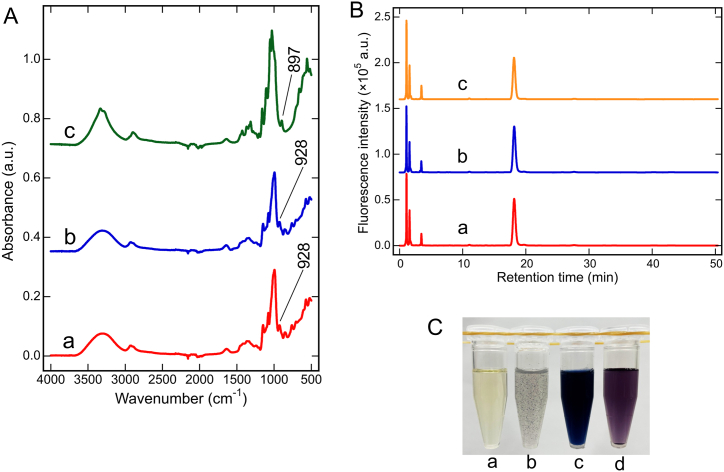
Chemical structure of the purified gel powder. (A) ATR-FTIR spectra of (a) purified sword bean gel powder, (b) soluble starch, and (c) cellulose. (B) Analysis of sugar composition of the sword bean gel. ABEE-labeled (a) gel powder, (b) soluble starch, and (c) glucose were applied to a reversed-phase C18 column using HPLC. (C) Starch–iodine reaction of (a) DW, (b) crude extract, (c) 3 % purified gel powder solution, and (d) 1 % soluble starch solution.

Sword beans contain carbohydrates and dietary fibers [[3], [4], [5]]. To deduce the chemical structure of substances in the gel powder, we compared the IR spectrum of the gel powder with those of starch and cellulose (Fig. 3A, spectra a–c, respectively). Although our previous study has proven that the gelling substance is not cellulose based on the differences in solubility in hot water [7], we also included cellulose in our analysis for a clear denial. The gel powder and starch presented similar spectral patterns, including a peak at 928 cm^−1^ derived from the skeletal vibrational mode of α-1,4 glycosidic linkages [[12], [13], [14], [15]]. However, the IR spectrum of the gel powder did not include the 896 cm^−1^ peak of cellulose that is characteristic of the β-glycoside linkage [[16], [17], [18], [19]]. ABEE-labeling was used to determine the constituent sugars in the gel powder hydrolysate, starch hydrolysate, and glucose (Fig. 3B, spectra a–c, respectively). According to the HPLC analysis, all ABEE-labeled samples presented a peak at the same retention time of 18.2 min, confirming that the constituent sugar of the gel powder was glucose. Moreover, the integrated peak areas indicated that the starch solution and gel powder solution contained similar amounts of glucose units (43.8 ± 3.3 and 40.6 ± 1.8 μg/mL, respectively, *p* = 0.134). Meanwhile, the total area of other peaks was sufficiently small (<1 %). From these results, we determined that the gel powder prepared from sword bean extract consisted of starch. The spectral agreement with starch further indicated that the prepared gelling substance had high purity.

In our previous study, the crude extract of sword beans was only slightly stained in the starch–iodine reaction, suggesting that the gelling substance was unlikely to be starch [7]. This does not agree with the chemical analyses described above. To resolve the discrepancy, the crude extract and powder solution prepared using the new extraction method were also subjected to the starch–iodine reaction (Fig. 3C). Again, the crude extract stained slightly (Fig. 3C–b), in agreement with our previous study [7]. By contrast, the gel powder solution was strongly stained (Fig. 3C–c), indicating that it was composed of starch and in agreement with the chemical analyses above. Nevertheless, the crude extract also presented several fairly dark granules suspended in the liquid (Fig. 3C–b), suggesting that (1) a part of the starch had aggregated with some impurities in the crude extract, and (2) the aggregated starch was in a stainable form. Notably, staining in the starch–iodine reaction is known to originate from the left-handed helical structure of amylose [20,21]. Generally, the blue color of amylose stained in the starch–iodine reaction fades when heated to a high temperature, and the blue color is restored after cooling [21]. It is also known that when amylose forms a complex with lipids and proanthocyanidin, the product is not stained by the starch–iodine reaction [22,23]. When amylose in the semi-flexible or worm-like chain state forms left-handed helices, chromogenic iodine species can become encapsulated in the helical structure and form hydrogen bonds with amylose to impart color [20,[24], [25], [26], [27]]. By contrast, absent or weak coloration in the starch–iodine reaction indicates that any starch in the sample exists in a form that cannot bind chromogenic iodine species [20,[24], [25], [26], [27]]. Therefore, the starch in our crude extract likely lost its helical structure during heating and reverted to its helical structure after cooling [20,24]. Notably, when the structure is restored, other substances in the crude gel may prevent coloration in the starch–iodine reaction [28]. Specifically, heating during extraction destroyed the helical structure of starch. After the crude extract cooled, the starch still could not revert to its helical structure owing to impurities. After washing the impurities off with DW, the starch restored itself to its helical structure and was capable of binding chromogenic iodine species. As the gel powder was purified from the crude extract, we speculated that starch in the crude extract had a molecular configuration that could not be stained, and it reverted to a stainable form after removing the impurities. This would reconcile all observations made in the previous and current studies.

In addition, we have previously reported that canavalin, the major protein of sword bean, can be isolated from crude sword bean extracts at low salt concentrations [29]. Because canavalin is also purified from the same crude extracts, it may lead to the misleading impression that different substances, that is, protein and starch, are extracted from the same crude extract. However, heating is not used in the extraction of canavalin [6,29], whereas it is used in the extraction of gelling substances [6,7]. Heating the crude extract is essential for extracting its gelling substance [7]. The crude extract contains protein, most of which is removed by heating and centrifugation [6]. This means that heating plays an important role in both extraction and purification.

### 3.6 Comparison of gelation behavior with other starches

**Fig. 4:**
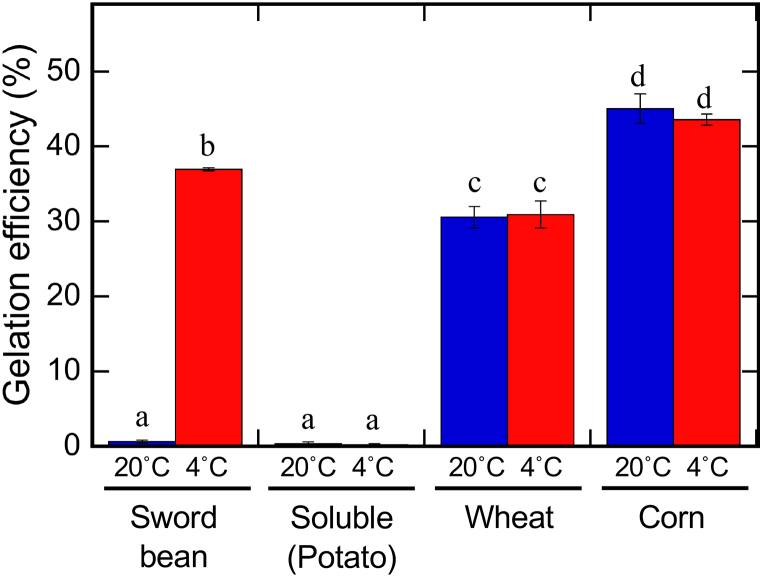
Gelation efficiency of the purified sword bean gel powder, soluble starch, wheat starch, and corn starch. The samples were dispersed at 3 % w/w. The dispersion was autoclaved as described in the experimental section. The gelation efficiency was calculated in the same way as depicted in Fig. 1A. Data are presented as the mean ± standard deviation of three independent replicates. The statistical significance of differences was determined by one-way analysis of variance and Tukey's honestly significant difference test. Different letters indicate statistically significant differences (*p* < 0.05).

We observed the gelation behavior of several typical starches under similar cooling conditions (Fig. 4). The starch solutions (3 %) were prepared in the same way as the sword bean gel powder solution. Soluble starch (amylodextrin produced from the hydrolysis of potato starch) did not gel when incubated at either 20 °C or 4 °C. After autoclaving, potato starch partially gelled immediately, making it impossible to prepare a uniform solution to assess its gelation efficiency. (Hence, the data are not shown.) By contrast, solutions of wheat and corn starches presented high gelation efficiencies when incubated at both 20 °C and 4 °C. The gelation efficiencies of these two starches also did not change significantly between 20 °C and 4 °C (wheat: *p* = 0.455, corn: *p* = 0.886). These results indicate that starch extracted from sword beans differs in its melting and gelling temperatures from potato, wheat, and corn starches and amylodextrin.

This sword bean gel powder may be useful in various processed foods, because its characteristic melting and gelling temperatures differ from those of commercially available gelling substances. Notably, corn starch and wheat starch, which, respectively, make up approximately 64 % and approximately 6 % of the world's starch production [30], both gel at 20 °C (Fig. 4). Potato starch, which makes up approximately another 6 % of the world's starch production [30], gels at room temperature immediately after autoclaving (data not shown). Meanwhile, when amylodextrin produced from the hydrolysis of potato starch was dissolved in DW by autoclaving, the solution did not gel at either 20 °C or 4 °C (Fig. 4). This inability of sword bean starch to gel at room temperature may improve the workability during food processing and cooking. Incidentally, a recent study reported the possibility of using sword bean starch as a material for biodegradable films [31]. Sword bean starch films containing goji berry (*Lycium barbarum* L.) extracts may serve as novel antioxidants to improve the shelf life of foods. Determining the detailed physical properties of sword bean starch may facilitate expanded uses of this material in not only processed foods but also biodegradable films in the food industry. Therefore, the amylose–amylopectin ratio, which is probably responsible for the characteristic gelling and melting temperatures, must be determined. This may require further purification using HPLC. In addition, viscosity and precise thermal properties, which are important for practical use, should also be investigated in future studies.

## 4 Conclusion

We developed an improved method using autoclaving to extract gelling substances from sword beans. The proposed extraction method exhibited almost twice the efficiency of a previously reported method [7]. The gelling substance was purified, and the obtained gel was converted to a gel powder; in total, 0.14 g of gel powder was obtained from 1 g of dried beans. The main constituent of the gelling substance was determined to be starch with unique gelling (12 °C) and melting temperatures (60 °C). We also determined the solubility limits of this gel powder (6 %) and its linear concentration range, which should enable further quantitative studies. Thus, our results can facilitate the use of sword bean-derived gel as a new ingredient in the food industry.

## Data availability statement

Data can be made available upon reasonable request to the corresponding author.

## Funding

This research did not receive any specific grant from funding agencies in the public, commercial, or not-for-profit sectors.

## CRediT authorship contribution statement

**Yasuhiro Arii:** Writing – review & editing, Writing – original draft, Visualization, Validation, Supervision, Software, Resources, Project administration, Methodology, Investigation, Funding acquisition, Formal analysis, Data curation, Conceptualization. **Kaho Nishizawa:** Writing – review & editing, Writing – original draft, Visualization, Methodology, Investigation, Formal analysis, Data curation.

## Declaration of competing interest

The authors declare no conflict of interest.
